# Hybrid surgery versus anterior cervical discectomy and fusion in multilevel cervical disc diseases: a meta-analysis: Retraction

**DOI:** 10.1097/MD.0000000000017483

**Published:** 2019-10-04

The article, “Hybrid Surgery Versus Anterior Cervical Discectomy and Fusion in Multilevel Cervical Disc Diseases: A Meta-Analysis”,^[1]^ which appears in Volume 95, Issue 21 of *Medicine*, is being retracted. The article appeared previously in Volume 5 of *Scientific Reports* as “Hybrid surgery versus anterior cervical discectomy and fusion for multilevel cervical degenerative disc diseases: a meta-analysis.”^[2]^

## References

[1] Zhgan J Meng F Ding Y Hybrid Surgery Versus Anterior Cervical Discectomy and Fusion in Multilevel Cervical Disc Diseases: A Meta-Analysis. *Medicine*. 95:e3621. 27227922 10.1097/MD.0000000000003621 PMC4902346

[2] Tian P Fu X Li Z-J Hybrid surgery versus anterior cervical discectomy and fusion for multilevel cervical degenerative disc diseases: a meta-analysis. *Sci Rep*. 5:13454. 10.1038/srep13454 PMC4549689 26307360

